# Borderline-resectable pancreatic adenocarcinoma: Contour irregularity of the venous confluence in pre-operative computed tomography predicts histopathological infiltration

**DOI:** 10.1371/journal.pone.0208717

**Published:** 2019-01-02

**Authors:** Georgios A. Kaissis, Fabian K. Lohöfer, Sebastian Ziegelmayer, Julia Danner, Carsten Jäger, Rebekka Schirren, Donna Ankerst, Güralp O. Ceyhan, Helmut Friess, Ernst J. Rummeny, Wilko Weichert, Rickmer F. Braren

**Affiliations:** 1 Institute for diagnostic and interventional Radiology, Klinikum rechts der Isar der Technischen Universität München, Munich, Germany; 2 Department of Surgery, Klinikum rechts der Isar der Technischen Universität München, Munich, Germany; 3 Department of Mathematics, Technische Universität München, Garching, Germany; 4 Department of General Pathology and Pathological Anatomy, Klinikum rechts der Isar der Technischen Universität München, Munich, Germany; University Hospital Hamburg Eppendorf, GERMANY

## Abstract

**Purpose:**

The purpose of the current study was to compare CT-signs of portal venous confluence infiltration for actual histopathological infiltration of the vein or the tumor/vein interface (TVI) in borderline resectable pancreatic ductal adenocarcinoma (PDAC).

**Methods and materials:**

101 patients with therapy-naïve, primarily resected PDAC of the pancreatic head without arterial involvement were evaluated. The portal venous confluence was assessed for contour irregularity (defined as infiltration) and degree of contact. The sensitivity and specificity of contour irregularity versus tumor to vein contact >180° as well as the combination of the signs for tumor cell infiltration of the vessel wall or TVI was calculated. Overall survival (OS) was compared between groups.

**Results:**

Sensitivity and specificity of contour irregularity for identification of tumor infiltration of the portal venous confluence or the TVI was higher compared to tumor to vessel contact >180° for tumor cell infiltration (96%/79% vs. 91%/38% respectively, p<0.001). The combination of the signs increased specificity to 92% (sensitivity 88%). Patients with contour irregularity/ tumor to vein contact >180°/ both signs had significantly worse overall survival (16.2 vs. 26.5 months/ 17.9 vs. 37.4 months/ 18.5 vs. 26.5 months respectively, all p<0.05).

**Conclusion:**

Portal venous confluence contour irregularity is a strong predictor of actual tumor cell infiltration of the vessel wall or the TVI and should be noted as such in radiological reports.

## Introduction

Pancreatic ductal adenocarcinoma remains one of the deadliest malignant conditions, partly due to its propensity for desmoplastic reaction and infiltration of the surrounding tissues, including perineural and vascular invasion [1]. Highest survival rates are reported for macroscopically total surgical removal of the tumor [2], which can be achieved in about 84% of patients that undergo surgery [3]. In an effort to increase the number of patients amenable for a curative treatment, surgical techniques have further evolved, with neoadjuvant chemotherapy regimens introduced and currently undergoing testing in phase II clinical trials (NEONAX, NCT02047513; NEOLAP, NCT02125136). Accordingly, radiographic signs of resectability have been adjusted to include tumors with vascular involvement and the potential for vascular resection and reconstruction, comprising “borderline resectable”, (BR) tumors. [4, 5].

Among the several accepted criteria for the definition of resectability in PDAC, the relationship of the tumor to the adjacent vasculature in pre-operative imaging is considered fundamental. Since the initial reports by Lu et al. [6] that classified degree of tumor contact (arterial and/or venous) ≥180° and by Hough et al. [7] that classified contour irregularity, i.e. *“teardrop sign”*, as indicative of non-resectability, several other definitions of arterial and venous resectability have been put forward by the respective societies. According to the most recent *NCCN* guidelines [8], the above-mentioned venous involvement is now explicitly included in BR. For the various imaging findings, differences in outcome have been reported, partly due to the inclusion of a broad range of imaging techniques, clinical stages, tumor locations and types of vascular involvement (e.g. arterial and venous) in the studies. Importantly, many studies lack a correlation of imaging findings with the most fundamental outcome parameter, histopathological infiltration of the interface between tumor and vein (TVI) and the vessel wall. However, with the introduction of promising neoadjuvant treatment plans, specific differentiation of tumor stages and an exact definition of tumor margins is needed. As PDAC patients are increasingly treated in specialized centers by expert clinicians, radiological reports should aim at the highest achievable precision. Thus, the rationale of the current study was to re-evaluate the predictive power of the CT-signs “contour irregularity” and “degree of tumor to vein-contact” [4] for histopathologically confirmed infiltration of the vein or the TVI in a therapy-naïve cohort of pancreatic head cancer patients who underwent tumor resection with curative intention.

## Materials and methods

### Ethics approval

The study was approved by the institutional ethics committee (Ethikkommission der Fakultät für Medizin der Technischen Universität München; 180/17 S) waiving the requirement for written informed consent for the retrospective data analysis.

### Study design

#### Patient cohort

A total of 312 patients who underwent pancreaticoduodenectomy between 07/2007 and 10/2014 were considered for inclusion in the study, and of these, 101 patients (54 male and 47 female) were included in the study and retrospectively analysed.

The decision for surgical exploration was made by an interdisciplinary tumor conference. The time period between acquisition of the pre-operative CT and surgical exploration was 2±1 weeks.

#### Exclusion criteria

Patients with evidence of metastatic spread at diagnosis, previous history of malignant disease, evidence of arterial involvement in pre-operative CT or early postoperative complications (Clavien-Dindo-Score ≥3 within 2 weeks after surgery) were excluded to limit any bias from early surgery-related complications on OS. No patient received neoadjuvant treatment. Patients who had undergone endoscopic intervention for the placement of a gall-duct stent were excluded because of potential imaging artifacts resulting from this intervention. Patients who had undergone endoscopic ultrasound or biopsy were not excluded.

#### Imaging parameters

CT data were included in the study if they met the following criteria: contrast enhanced venous phase in inspiratory breath-hold with portal vein attenuation ≥ 140 Hounsfield units, slice thickness at most 3mm, availability of axial, coronal and sagittal multiplanar reformations, depiction of the body from the pulmonary apex to the proximal femurs. Data were acquired with 16- to 64-row CT-scanners and reconstructed using medium-hard kernels.

#### Radiological observers

All images were initially reviewed independently by 3 radiologists blinded to the histopathological outcome (GK, FL, RB with 4, 6 and 10 years of experience, respectively). A consensus was reached for all images with the most experienced radiologist (RB) serving as tie-breaker in cases of disagreement (n = 8 cases). Images were reviewed under standardized lighting conditions using the same hospital PACS workstation running SECTRA IDS 7.

#### Definition of CT-signs of near portal venous confluence infiltration

**Fig 1:**
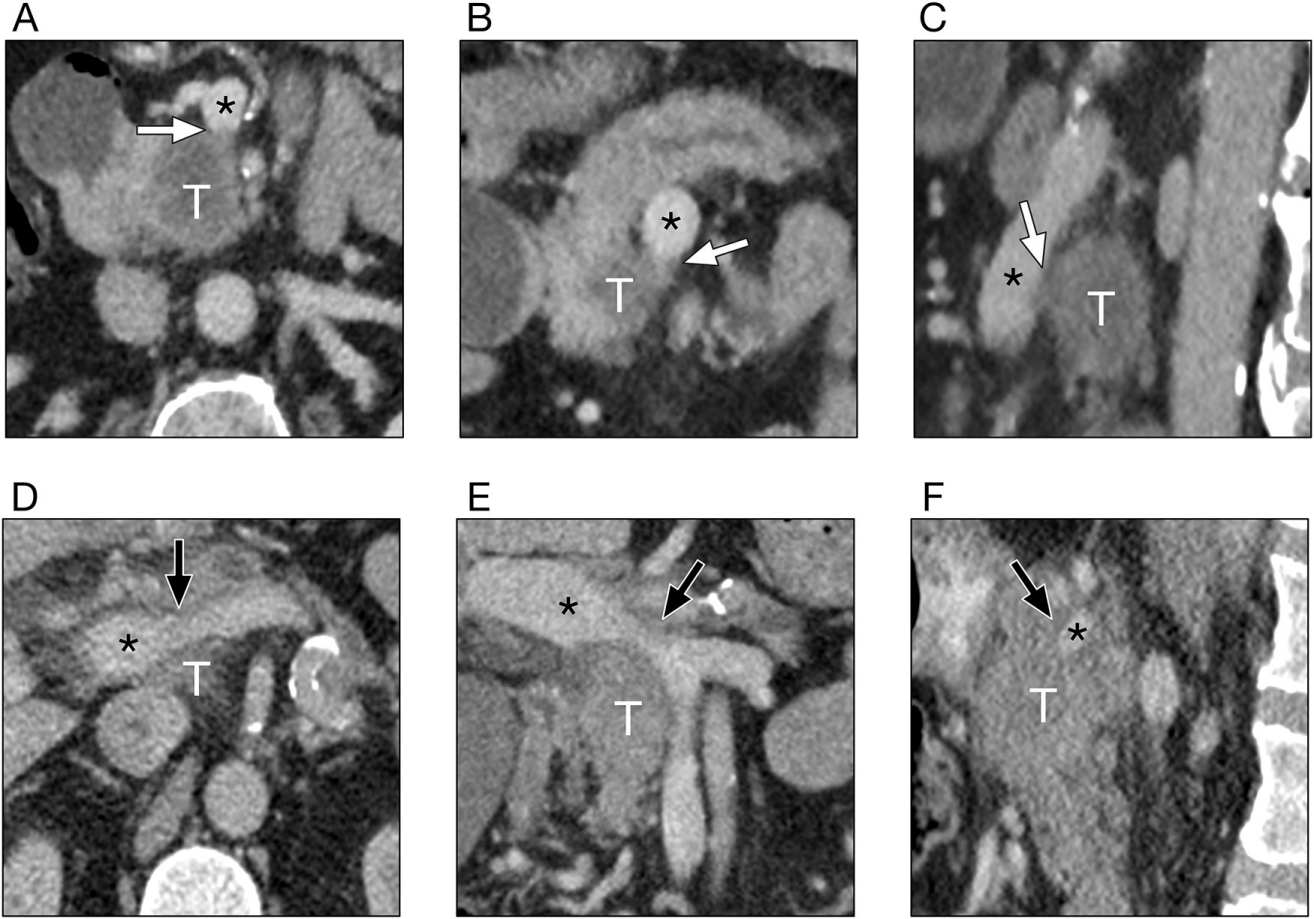
Venous contour irregularity witnessed in two patients. A-C show soft tissue distorting the vessel (white arrows). D-F show focal vessel narrowing (black arrows). Tumor (T) and portal vein (*) are marked accordingly. All images taken from 3mm multiplanar reformation in the axial (A/D), coronal (B/E) and saggital (C/F) planes.

The portal vein, defined as the vessel proximal to the confluence of the superior mesenteric and splenic veins, and the superior mesenteric vein up to 2 cm distal to the confluence were assessed in all 3 multiplanar reformations. Portal venous confluence infiltration was defined as vessel contour irregularity according to current radiological reporting recommendations [4] (Fig 1). Degree of contact between solid tumor and the portal venous confluence was judged ≤180° versus >180°.

#### Surgical procedure

All patients underwent pylorus-sparing pancreatic head resection in the same seating after surgical exploration. The decision for or against venous resection was made by the surgeon according to intraoperative impression of venous infiltration.

#### Histopathological analysis

Definitive surgical pathology reports signed by 2 subspecialized pathologists were reviewed. Maximal tumor size, pTNM classification and tumor grading (UICC 8^th^ ed.) were recorded. Vessel wall infiltration to the level of the adventitia or deeper, or tumor cell presence at or ≤1mm from the interface between the tumor and the vein was explicitly identified and classified as present or absent in the report.

#### Statistical analysis

All statistical tests were performed using GraphPad Software’s *Prism 7*. A significance level of p<0.05 was defined. The sensitivity, specificity, positive and negative predictive values of portal venous confluence contour irregularity and for degree of contact between the tumor and the portal venous confluence >180° for prediction of actual tumor cell infiltration of the venous wall or the TVI were calculated using Fisher’s exact test. The Chi-Square-Test was used for cross-table analysis. Survival comparisons were performed using a Mantel-Cox log-rank test, after ascertaining that model assumptions were met.

## Results

Average age at diagnosis was 69.5±11.2 years, with tumor size between 25 and 40mm (maximum dimension as stated in the final histopathological report). All patients had locoregional lymph node spread. 94 patients received adjuvant chemotherapy with Gemcitabine following interdisciplinary tumor board decision (of these, 3 patients received additional Cetuximab, Erlotinib or Capecitabine) while 7 patients denied adjuvant chemotherapy. Tumor grading was G1 in 9 cases, G2 in 46 cases and G3 in 46 cases.

The distribution of tumor grading and adjuvant chemotherapy regimen for patients with and without venous contour irregularity and with tumor to vein contact >180°/ ≤180° are shown in Tables 1 and 2 respectively.

**Table 1:** Distribution of tumor grading and adjuvant chemotherapy regimens is shown for the patient cohorts with/ without venous contour irregularity.

	Venous contour irregularity (N = 62)	No venous contour irregularity (N = 39)	Chi-Squared-Test p
Grading	G1: 4 (6.6%) G2:29 (46.7%) G3:29 (46.7%)	G1: 5 (12.8%) G2: 17 (43.6%) G3: 17 (43.6%)	0.54
Adjuvant Chemotherapy	Received Therapy: 58 (93.5%) Denied Therapy: 4 (6.5%)	Received Therapy: 36 (92.3%) Denied Therapy: 3 (7.7%)	0.81

**Table 2:** Distribution of tumor grading and adjuvant chemotherapy regimens is shown for the patient cohorts with tumor to vein contact ≤180°/>180°.

	Tumor to vein contact >180° (N = 78)	Tumor to vein contact ≤180° (N = 23)	Chi-Squared-Test p
Grading	G1: 6 (7.7%) G2:38 (48.7%) G3:34 (43.6%)	G1: 3 (13.0%) G2: 8 (34.8%) G3: 12 (52.2%)	0.44
Adjuvant Chemotherapy	Received Therapy: 72 (92.3%) Denied Therapy: 6 (7.7%)	Received Therapy: 22 (95.7%) Denied Therapy: 1 (4.3%)	0.57

**Fig 2:**
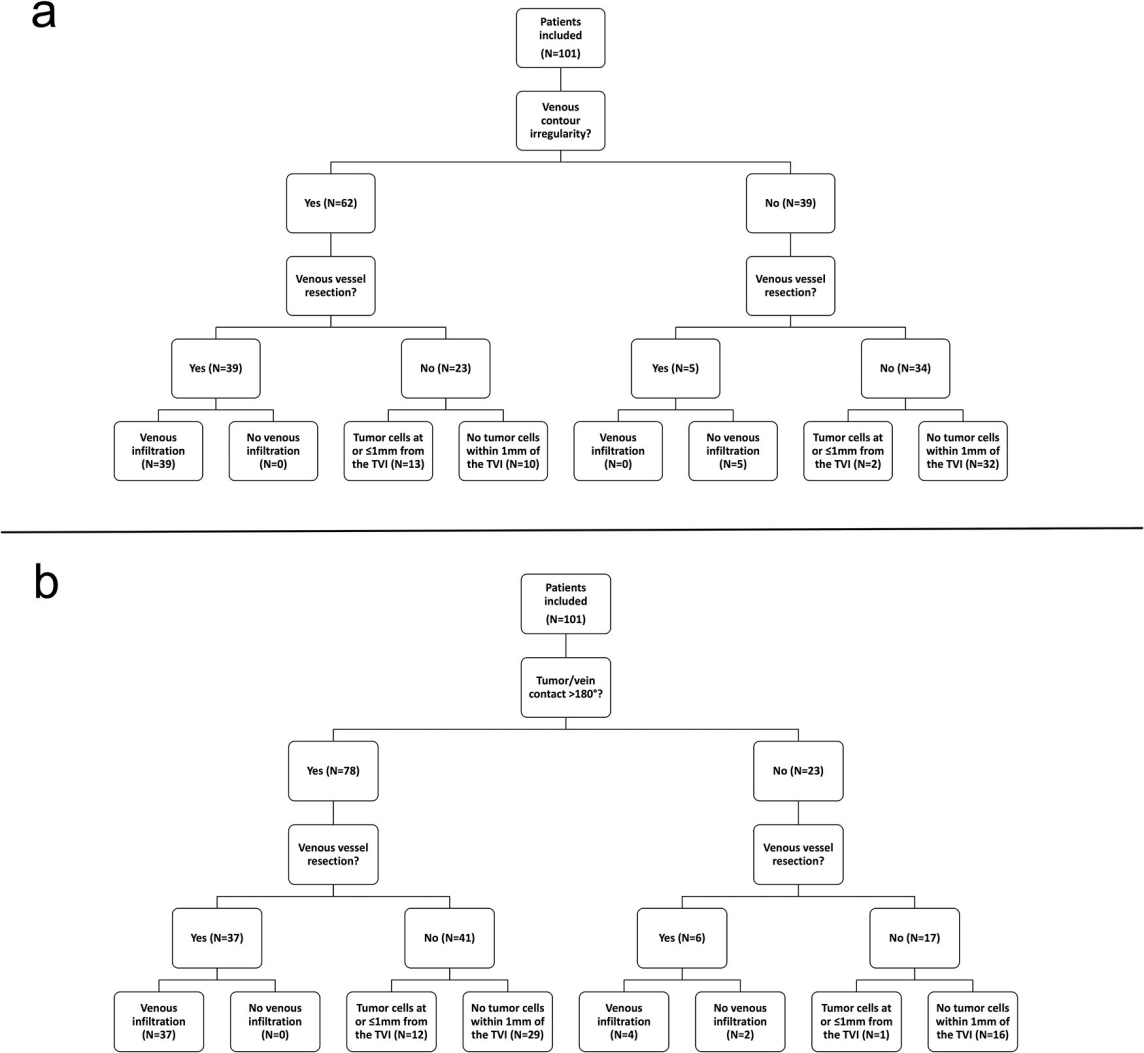
Flowchart showing distribution of patients in groups with and without venous contour irregularity (a) and patients in groups with tumor to vein contact >180° or ≤180° (b) as well as results of histopathological examination.

Portal venous confluence contour irregularity and degree of tumor to vessel-contact on pre-operative CT as well as histopathology specimens of 101 patients that underwent pancreatic head resection for PDAC were reviewed (Fig 2a).

Of these, 62 patients (61.4%) exhibited a contour irregularity of the portal venous confluence. Thereof, 39 patients (62.9%) underwent venous resection and all of these revealed tumor cell infiltration of the vessel wall at histopathological analysis of the resection specimen. Of the remaining 23 patients who did not undergo venous resection, 13 (56.5%) revealed tumor cell infiltration at or ≤1mm from the TVI. In contrast, of the 39 patients who did not show a contour irregularity, 5 (12.8%) underwent venous resection and none (0%) of them exhibited tumor cell infiltration of the vessel wall or ≤1mm from the TVI. Of the remaining 34 patients who showed no contour irregularity and did not undergo venous resection, only 2 (5.9%) had tumor cells ≤ 1mm from the TVI.

In comparison, tumor to vessel contact >180° was noted in 78 (77.2%) of the 101 patients. Thereof, 37 patients (47.4%) underwent venous resection and all (100%) exhibited tumor cell infiltration of the vessel wall on histopathological analysis. Of the remaining 41 patients, only 12 (29.3%) exhibited tumor cell infiltration at or ≤1 mm from the TVI. Furthermore, of the 23 patients with tumor to vessel contact ≤180°, a total of 5 (21.7%) exhibited either tumor cell infiltration of the vessel wall (n = 4) or at or ≤1mm from the TVI (n = 1) (Fig 2b).

**Fig 3:**
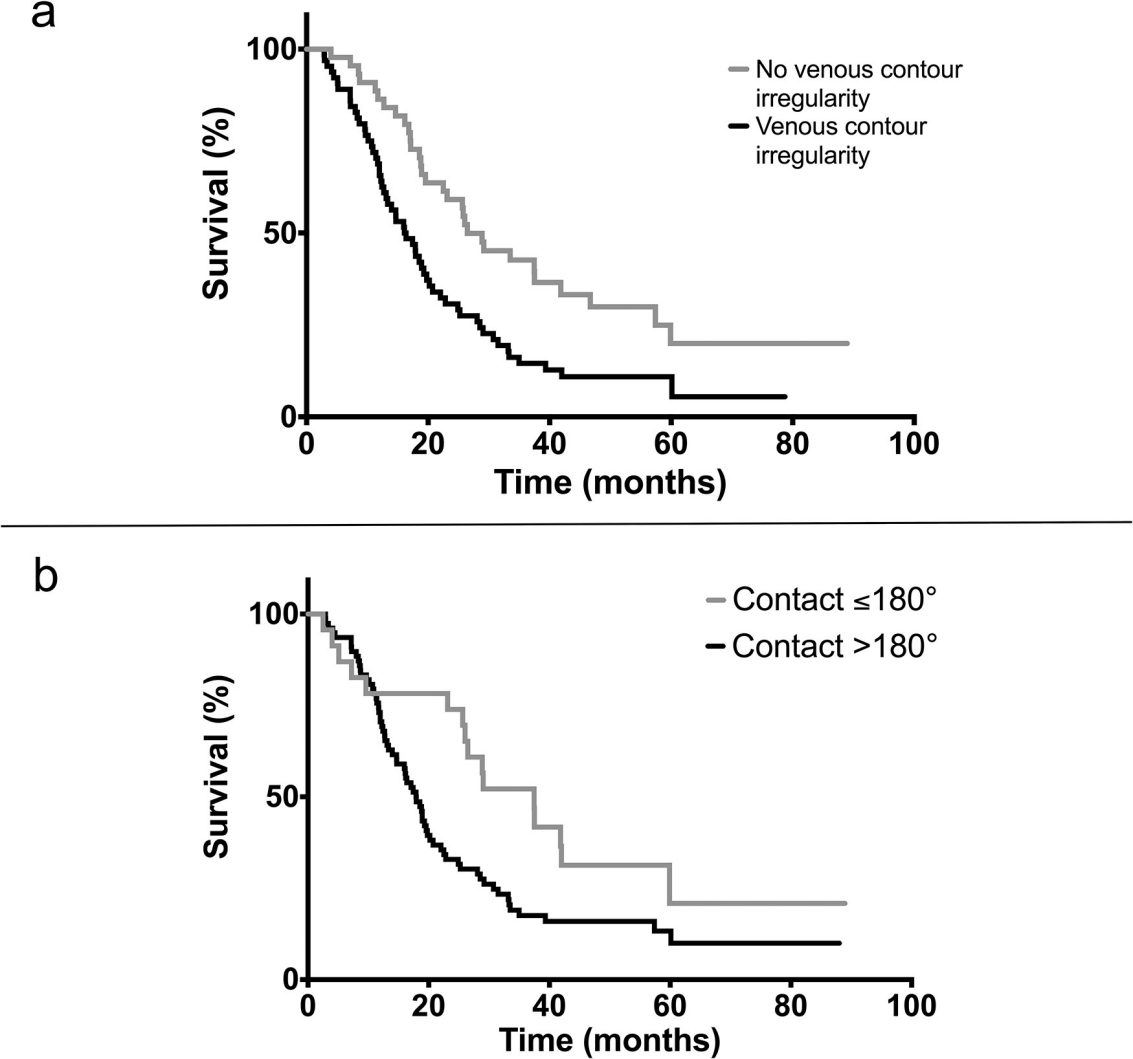
Kaplan-Meier survival curves of patients with venous contour irregularity and without venous contour irregularity (a). Kaplan-Meier survival curves of patients with tumor to vein contact ≤180° and >180° (b).

Median OS in patients with or without venous confluence contour irregularity was 16.2 months and 26.5 months, respectively (n = 101, p = 0.001) (Fig 3a).

Patients with tumor to vein contact >180° had a median OS of 17.9 months vs. 37.4 months in the group with tumor to vein contact ≤180° (n = 101, p = 0.02), although the survival curves crossed, indicating violation of the proportional hazards model that should thus be interpreted with care (Fig 3b).

A total of 13/101 (12.8%) of cases were censored.

Contour irregularity vs. tumor to vessel contact >180° showed a higher sensitivity (96.3% vs. 90.7%), specificity (78.7% vs. 38.3%) as well as positive (83.9% vs 62.8%) and negative (94.8% vs 78.2%) predictive value for the histopathological outcome of tumor cell infiltration of the vessel wall or the TVI (p<0.0001 in both cases, total n = 101).

An analysis of the subgroup of patients who showed both venous contour irregularity and tumor to vessel contact >180° (n = 17, 13 of which showed signs of infiltration of the vessel wall, 1 showed signs of infiltration of the TVI and 3 showed neither signs of infiltration of the vessel wall or the TVI) vs. patients who showed neither of the signs (n = 37, of which 35 showed no sign of infiltration of the vessel wall or the TVI and 2 showed signs of infiltration of the TVI but none with actual vessel wall infiltration) yielded a sensitivity of 87.5%, a specificity of 92.1%, a positive predictive value of 82.4% and a negative predictive value of 94.6%. (Table 3).

**Table 3:** Sensitivity and specificity, positive and negative predictive values of the signs of venous contour irregularity and contact to vein >180° as well as the combined analysis.

	Sensitivity	Specificity	Positive predictive value	Negative predictive value
**Venous contour irregularity**	96.3%	78.7%	83.9%	94.8%
**Contact to vein>180°**	90.7%	38.3%	62.8%	78.2%
**Venous contour irregularity AND contact to vein>180°**	87.5%	92.1%	82.4%	94.6%

**Fig 4:**
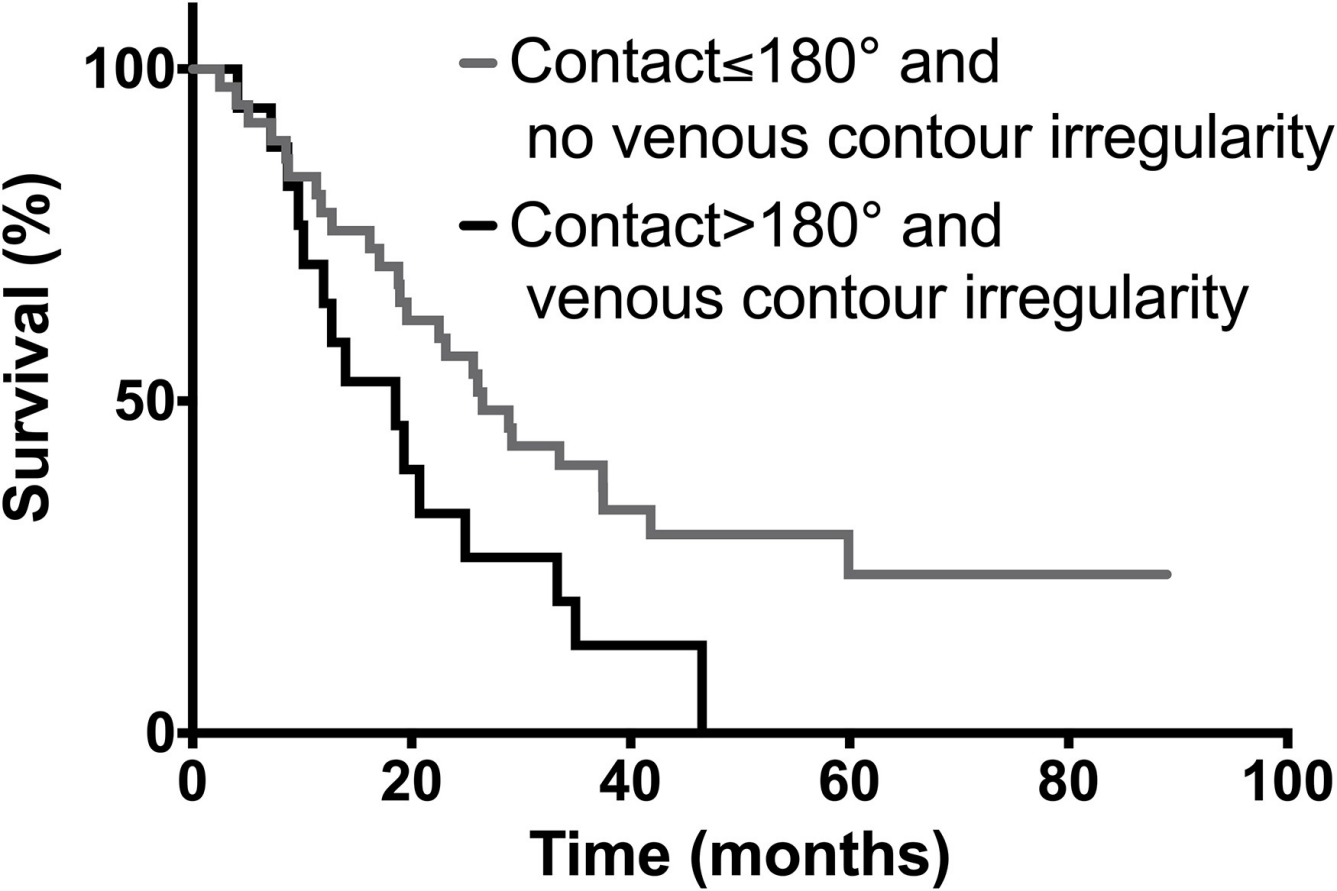
Kaplan-Meier survival curves of patients with both venous contour irregularity and contact to vein>180° and without either venous contour irregularity or contact to vein.

A Kaplan-Meier survival analysis of the combined analysis is seen in Fig 4. The median OS for patients with both venous contour irregularity and contact to vein>180° was 18.5 months, for patients without either venous contour irregularity or contact to vein >180° was 26.5 months (n = 17 vs. n = 37, p = 0.028).

## Discussion

Precise pre-therapeutic staging is of high importance in PDAC because patients are stratified into potentially curative versus palliative therapy regimens based on minute differences in tumor spread, which are best revealed on cross-sectional imaging. Among these, venous infiltration is an important finding that warrants vessel resection and replacement to ensure complete tumor removal in therapy-naïve patients. The present study demonstrates that venous confluence contour irregularity is a strong predictor of histopathologically identified tumor cell infiltration of the TVI or the vessel wall and of worse overall survival and that venous contour irregularity outperforms tumor to vein contact >180°.

Study results highlight the importance of identifying contour irregularity, which is and should remain a reporting standard in PDAC. Beyond the increased sensitivity of venous contour irregularity vs. tumor to vein contact, a combination of both signs yields increased specificity, thus we encourage denoting the presence of both the findings in radiological reports. The need for additional image review by subspecialized radiologists (e.g. “referral centers”) in cases of suspected or confirmed PDAC is underscored by the often-subtle nature of these radiographic findings. Our study only included CT data with availability of ≤3mm-thickness slices and three-plane MPRs, believing this to be the minimum requirement for accurate diagnosis and being in line with current literature [9], although we support aiming for the highest achievable spatial resolution by utilizing as thin as possible isotropic datasets for diagnosis. With advancements in CT technology, higher availability of custom MPRs “on the fly” as well as curved MPRs and 3D-rendering techniques are expected to further enhance sensitivity.

The high sensitivity of portal venous confluence contour irregularity observed in pre-operative CT for histopathological tumor cell infiltration of the TVI or vessel wall infiltration indicates that, even in cases where dissection of the vessel away from the tumor is technically possible without vessel removal, there is a high likelihood of tumor infiltration near the vein. The finding of venous contour irregularity thus supports an intensified (neo-)adjuvant chemotherapeutic approach and should be taken into consideration for patients who undergo primary resection. In the neoadjuvant setting, however, further investigation of the significance of venous contour irregularity is needed, since even non-vital tumor matrix can cause persistent vascular irregularity. In some cases, we were able to observe that in cases of a mismatch between the radiologist’s finding of venous contour irregularity and the intraoperative impression (i.e. the surgeon suspecting venous infiltration and resecting the vessel while no contour irregularity was observed), the lack of contour irregularity seems to be a better predictor of the histopathological outcome, although this should be interpreted with care in this very small subcohort of 5 cases.

The diagnostic sensitivity and specificity in our study is higher than those reported in a previous study including a portion of the same cohort [10]. However, this prior study included only 23 patients, imaging studies included both CT and MRI, no exclusion of cases because of image quality was undertaken, and patient selection was not as restrictive as in the present study.

In our patient cohort, venous contour irregularity correlated with a worse overall survival. This finding is not surprising in this historical cohort without neoadjuvant therapy and falls in line with other studies [11] which found invasion of the portal or superior mesenteric vein to be an independent predictor of worse overall survival. With continuously improving surgical technique and inclusion of more patients in neoadjuvant therapy regimes, we expect current patient cohorts with venous infiltration to show improved survival as demonstrated in a current Korean study [12].

We undertook care to eliminate sources of bias with regards to clinical characteristics. Since the UICC system has changed concerning T-stage of PDAC from the 7^th^ to the 8^th^ edition, we decided to select patient cases based on actual tumor diameter and to forgo “T”-classification entirely. Furthermore, all patients had N1 nodal status. The distribution of adjuvant chemotherapy and tumor grading was balanced in the examined cohorts to a degree we do not expect to significantly skew results. No patient included in this study received neo-adjuvant chemotherapy. Therefore, the reported findings need to be re-evaluated in the setting of neo-adjuvant chemotherapy as the finding of venous confluence contour irregularity may not have the same implications under such conditions.

Our study has several limitations. As a retrospective single-center study, results may be skewed due to operative technique and preference as well as departmental-specific radiological reporting. Although the overall cohort size was 101 patients, histopathological data on venous infiltration was available for a subsample of 44 patients. Furthermore, although consensus was high, no calculation of inter-observer variability scores was performed. In non-specialized centers, rates of reporting might be lower due to lower exposure of radiologists to pancreatic cancer cases. The restrictive patient sampling we undertook to limit bias from tumor size or from variations in the spatial relationship between the position of the tumor and the vessels does not allow significant conclusions concerning any possible predilection areas for tumor infiltration along the vessels in question, which would need further evaluation in future studies.

Our study demonstrates that venous confluence contour irregularity is a sensitive and specific sign of tumor cell infiltration of the vessel wall or the nearby tissue, outperforming >180° tumor to vein-contact. It further shows that contour irregularity is a predictor of worse overall survival in this non-pretreated cohort. It underscores the importance of obtaining high quality pre-operative images that should be reviewed by experienced/ subspecialized radiologists. Attention to and inclusion of this -often subtle- finding in the standardized radiological report can lead to improved therapy, either through inclusion in neoadjuvant therapy regimens or through surgical resection of the vein.
